# ADSCs stimulated by resistin promote breast cancer cell malignancy via CXCL5 in a breast cancer coculture model

**DOI:** 10.1038/s41598-022-19290-6

**Published:** 2022-09-14

**Authors:** Yen-Yun Wang, Amos C. Hung, Yi-Chia Wu, Steven Lo, Huan-Da Chen, Yuk-Kwan Chen, Ya-Ching Hsieh, Stephen Chu‐Sung Hu, Ming-Feng Hou, Shyng-Shiou F. Yuan

**Affiliations:** 1 School of Dentistry, College of Dental Medicine, Kaohsiung Medical University, Kaohsiung, Taiwan; 2 Department of Medical Research, Kaohsiung Medical University Hospital, Kaohsiung, Taiwan; 3 Graduate Institute of Medicine, College of Medicine, Kaohsiung Medical University, Kaohsiung, Taiwan; 4 School of Medicine, College of Medicine, Kaohsiung Medical University, Kaohsiung, Taiwan; 5 Division of Plastic Surgery, Department of Surgery, Kaohsiung Medical University Hospital, Kaohsiung, Taiwan; 6 Division of Breast Oncology and Surgery, Department of Surgery, Kaohsiung Medical University Hospital, Kaohsiung, Taiwan; 7 Regenerative Medicine and Cell Therapy Research Center, Kaohsiung Medical University, Kaohsiung, Taiwan; 8 College of Medical, Veterinary and Life Sciences, University of Glasgow, Glasgow, UK; 9 Translational Research Center, Kaohsiung Medical University Hospital, Kaohsiung, Taiwan; 10 Division of Oral Pathology and Maxillofacial Radiology, Kaohsiung Medical University Hospital, Kaohsiung, Taiwan; 11 Oral and Maxillofacial Imaging Center, College of Dental Medicine, Kaohsiung Medical University, Kaohsiung, Taiwan; 12 Institute of Cancer Sciences, University of Glasgow, Glasgow, UK; 13 Department of Dermatology, College of Medicine, Kaohsiung Medical University, Kaohsiung, Taiwan; 14 Department of Dermatology, Kaohsiung Medical University Hospital, Kaohsiung, Taiwan; 15 Department of Biomedical Science and Environmental Biology, College of Life Science, Kaohsiung Medical University, Kaohsiung, Taiwan; 16 Department of Obstetrics and Gynecology, Kaohsiung Medical University Hospital, Kaohsiung, Taiwan; 17 Department of Biological Science and Technology, College of Biological Science and Technology, National Yang Ming Chiao Tung University, Hsinchu, Taiwan; 18 Center for Intelligent Drug Systems and Smart Bio-devices (IDS2B), National Yang Ming Chiao Tung University, Hsinchu, Taiwan

**Keywords:** Cancer, Oncology

## Abstract

The tumor microenvironment represents one of the main obstacles in breast cancer treatment owing to the presence of heterogeneous stromal cells, such as adipose-derived stem cells (ADSCs), that may interact with breast cancer cells and promote cancer development. Resistin is an adipocytokine associated with adverse breast cancer progression; however, its underlying mechanisms in the context of the breast tumor microenvironment remain largely unidentified. Here, we utilized a transwell co-culture model containing patient-derived ADSCs and breast cancer cell lines to investigate their potential interaction, and observed that breast cancer cells co-cultured with resistin-treated ADSCs (R-ADSCs) showed enhanced cancer cell growth and metastatic ability. Screening by proteome arrays revealed that C-X-C motif chemokine ligand 5 (CXCL5) was released in the conditioned medium of the co-culture system, and phosphorylated ERK was increased in breast cancer cells after co-culture with R-ADSCs. Breast cancer cells treated with the recombinant proteins of CXCL5 showed similarly enhanced cell migration and invasion ability as occurred in the co-culture model, whereas application of neutralizing antibodies against CXCL5 reversed these phenomena. The orthotopic xenograft in mice by breast cancer cells after co-culture with R-ADSCs had a larger tumor growth and more CXCL5 expression than control. In addition, clinical analysis revealed a positive correlation between the expression of resistin and CXCL5 in both tumor tissues and serum specimens of breast cancer patients. The current study suggests that resistin-stimulated ADSCs may interact with breast cancer cells in the tumor microenvironment via CXCL5 secretion, leading to breast cancer cell malignancy.

## Introduction

Breast cancer is the most commonly diagnosed cancer and the leading cause of cancer death in women according to GLOBOCAN estimates for 2020^1^. Among the risk factors that contribute to the development of breast cancer, obesity is a significant factor known to correlate with adverse breast cancer progression and poor treatment outcome in postmenopausal patients^2^. Obese adipose tissue may create a pro‐oncogenic environment by releasing a variety of cytokines, adipocytokines, chemokines, lipids, and growth-related factors in favor of tumor survival^3^. More recent research has proposed that some adipocytokines, such as visfatin and resistin, promote breast cancer progression through cell–cell interactions within the breast tumor microenvironment^4,5^.

Resistin is a secretory protein composed of 114 amino acids in mice or 108 amino acids in humans with 59% identity^6,7^. It is named after the initially characterized function of ‘resistance to insulin’ in obese mouse models, and is mainly secreted by mouse adipocytes^6^. Later studies revealed that resistin is also secreted by human adipose tissue-associated stromal cells^8–10^, suggesting that it has diverse functions across different species. Emerging lines of evidence have pointed to the role of human resistin as an important mediator in a number of obesity and inflammation-associated pathological conditions, including various cancers^4,11^. In particular, our group and others have noted that elevated resistin expression is associated with advanced breast tumor characteristics, such as increased tumor stages and lymph node metastasis in breast cancer patients^12–15^. However, the biological mechanisms underlying these phenomena remain to be explored.

The breast tumor microenvironment is a heterogeneous niche composed of tumor cells and multiple stromal cells with a significant proportion of adipose tissue-derived cells, which have been considered as key players in promoting the malignancy of breast cancer^16^. For example, co-culture of breast cancer cells with mouse adipocytes in a transwell model increased proliferation and migration ability of breast cancer cells^17^. In addition, breast cancer cells treated with the conditioned medium from human adipose tissue-derived macrophages exhibited enhanced malignant phenotypes, and these macrophages over-expressed a cluster of cancer-associated genes, with the C-X-C chemokine ligand 1 (CXCL1) and C-X-C chemokine ligand 5 (CXCL5) being mostly upregulated^18^. Moreover, dysregulation of adipose tissue-derived stem cells (ADSCs) has been associated with breast cancer progression and metastasis, possibly via the expression of multiple C-X-C chemokines^19–21^. As these adipose tissue-derived cells may have crucial roles in promoting breast malignancy by interacting with cancer cells in the breast tumor microenvironment, identifying and targeting their pathogenic molecular pathways have emerged as a potential strategy for breast cancer treatment^22,23^.

In this study, we examined the impact of resistin on ADSCs in promoting breast cancer cell malignancy, with an emphasis on their interaction with breast cancer cells in the context of the tumor microenvironment. To achieve this goal, a transwell co-culture model of resistin-stimulated ADSCs, which was derived from breast cancer patients, with human breast cancer cell lines was established for in vitro and in vivo investigation. Utilizing a cytokine/chemokine array to screen the secretory factors and a phospho-kinase array to search for signaling pathways involved in the co-culture model, we found that CXCL5, a chemokine that can promote breast tumor growth and metastasis^24–30^, played an important role in relaying the effect of resistin-stimulated ADSCs on breast cancer cell malignancy with the participation of ERK pathway. The clinical relevance of these cancer-promoting molecules was further evaluated through investigation of the protein expression in tumor tissues and serum specimens from breast cancer patients.

## Results

### Resistin-stimulated ADSCs enhanced malignant behaviors of breast cancer cells

**Figure 1:**
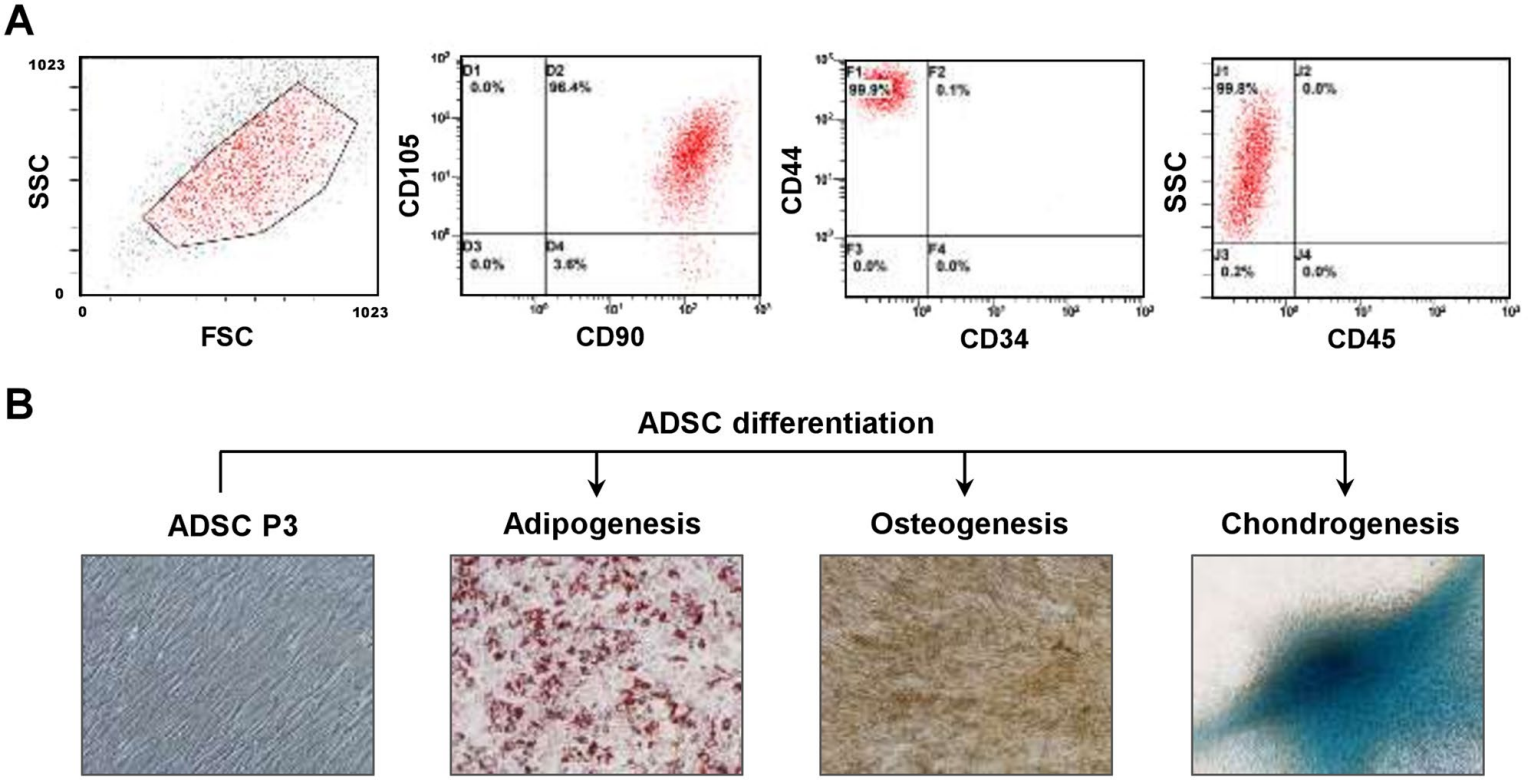
Characterization of isolated ADSC phenotypes and the potential for lineage-specific differentiation. (**A**) The ADSCs isolated from adipose tissues adjacent to breast tumors were characterized by the expression of mesenchymal stem cell markers (CD44, CD90, and CD105) or hematopoietic lineage markers (CD34 and CD45). After culturing for three passages, the ADSCs showed the expression of CD44^+^/CD90^+^/CD105^+^/CD34^–^/CD45^–^ cell surface markers which were analyzed by flow cytometry. (**B**) Representative images showed the undifferentiated ADSCs at passage 3 (ADSC P3), and these ADSCs were able to be induced to differentiate into three different cell lineages: adipocytes (adipogenesis), osteocytes (osteogenesis), and chondrocytes (chondrogenesis).

We first isolated primary ADSCs from breast tumor-adjacent adipose tissues in breast cancer patients with surgical treatment. Flow cytometric analysis showed that the isolated ADSCs expressed the mesenchymal stem cell markers CD44, CD90, and CD105, while they were deficient in the hematopoietic lineage markers CD34 and CD45 after culturing for three passages (Fig. 1A), which were in concordance with previous reports^31,32^. In addition, differentiation assays showed that the isolated ADSCs were able to differentiate into adipocytes, osteocytes, and chondrocytes (Fig. 1B). These results suggest that the isolated ADSCs possessed mesenchymal stem cell properties.

**Figure 2:**
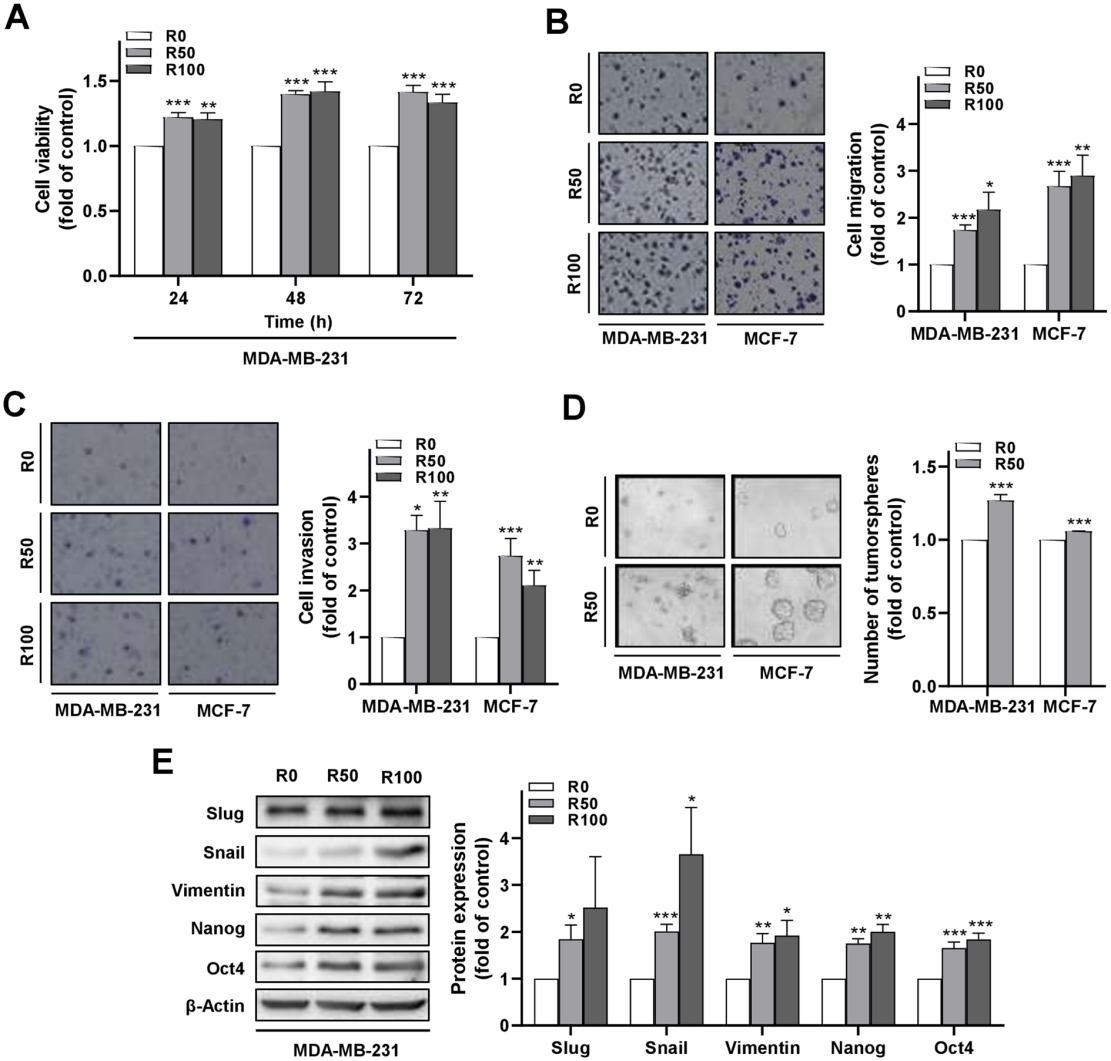
Enhanced malignant behaviors of breast cancer cells after co-culture with resistin-stimulated ADSCs. The isolated ADSCs were treated with resistin at 0, 50, and 100 ng/ml (R0, R50, and R100, respectively) for 48 h, followed by co-culture with MDA-MB-231 or MCF-7 cells in the transwell model for another 72 h before the following analyses. (**A**) MDA-MB-231 cells after co-culture with R-ADSCs (R50 and R100) or control ADSCs (R0) were collected and re-plated in 96-well plates for cell viability assay. (**B**) MDA-MB-231 or MCF-7 cells after co-culture with R-ADSCs (R50 and R100) or control ADSCs (R0) were collected and re-plated in the transwell inserts for cell migration assay. (**C**) MDA-MB-231 or MCF-7 cells after co-culture with R-ADSCs (R50 and R100) or control ADSCs (R0) were collected and re-plated in the transwell inserts coated with Matrigel for cell invasion assay. (**D**) MDA-MB-231 or MCF-7 cells after co-culture with R-ADSCs (R50) or control ADSCs (R0) were collected and re-plated in ultra-low attachment microplates for tumorsphere formation assay. Quantitation was carried out for those with diameter over 50 μm. (**E**) MDA-MB-231 cells after co-culture with R-ADSCs (R50 and R100) or control ADSCs (R0) were collected and analyzed for protein expression of mesenchymal markers (Slug, Snail, and Vimentin) and cancer stemness markers (Nanog and Oct4) by Western blot. The original blot images were available in Supplementary Information, and these blots were cut from full-length membranes prior to hybridization with the corresponding antibodies. Data were obtained from three independent experiments and presented as mean ± SEM. Statistical difference was determined by t-test comparing R50 or R100 group versus their corresponding R0 group as control. *p < 0.05; **p < 0.01; ***p < 0.001.

The isolated ADSCs were subsequently treated with resistin (R-ADSCs), or without resistin treatment as control, followed by transwell co-culture with human breast cancer cell lines for further investigation of the prolonged effects of resistin (Supplementary Fig. S1). The concentration of resistin applied to ADSCs in this study was in a range covering human resistin levels detected in the serum of breast cancer patients^15^. We observed an enhancement of cell viability in the group of MDA-MB-231 cells after co-culture with R-ADSCs compared with the control group (Fig. 2A). In addition, the extent of transwell cell migration and invasion was increased when MDA-MB-231, or a less aggressive breast cancer cell line MCF-7, underwent the co-culture condition with R-ADSCs (Fig. 2B,C). Moreover, the number of tumorsphere formation was increased after co-culture of these breast cancer cells with R-ADSCs, although the morphology of MDA-MB-231 showed flattened aggregation instead of round aggregates observed in MCF-7 (Fig. 2D). The colony formation in soft agar was also increased in MDA-MB-231 cells after co-culture with R-ADSCs (Supplementary Fig. S2). We further analyzed the protein level of epithelial-to-mesenchymal transition (EMT) and cancer stemness markers in MDA-MB-231 cells under the co-culture condition. As shown in Fig. 2E, the mesenchymal markers, such as Slug, Snail, and Vimentin^33^, and stemness markers, such as Nanog and Oct4^34^, were increased in MDA-MB-231 cells after co-culture with R-ADSCs. These data collectively suggest that resistin treatment had an impact on the activation of ADSCs, which resulted in the enhanced malignant behaviors of breast cancer cells, such as cancer cell growth and metastatic ability.

### Secreted CXCL5 from the co-culture of resistin-stimulated ADSCs and breast cancer cells enhanced breast cancer cell malignant behaviors

**Figure 3:**
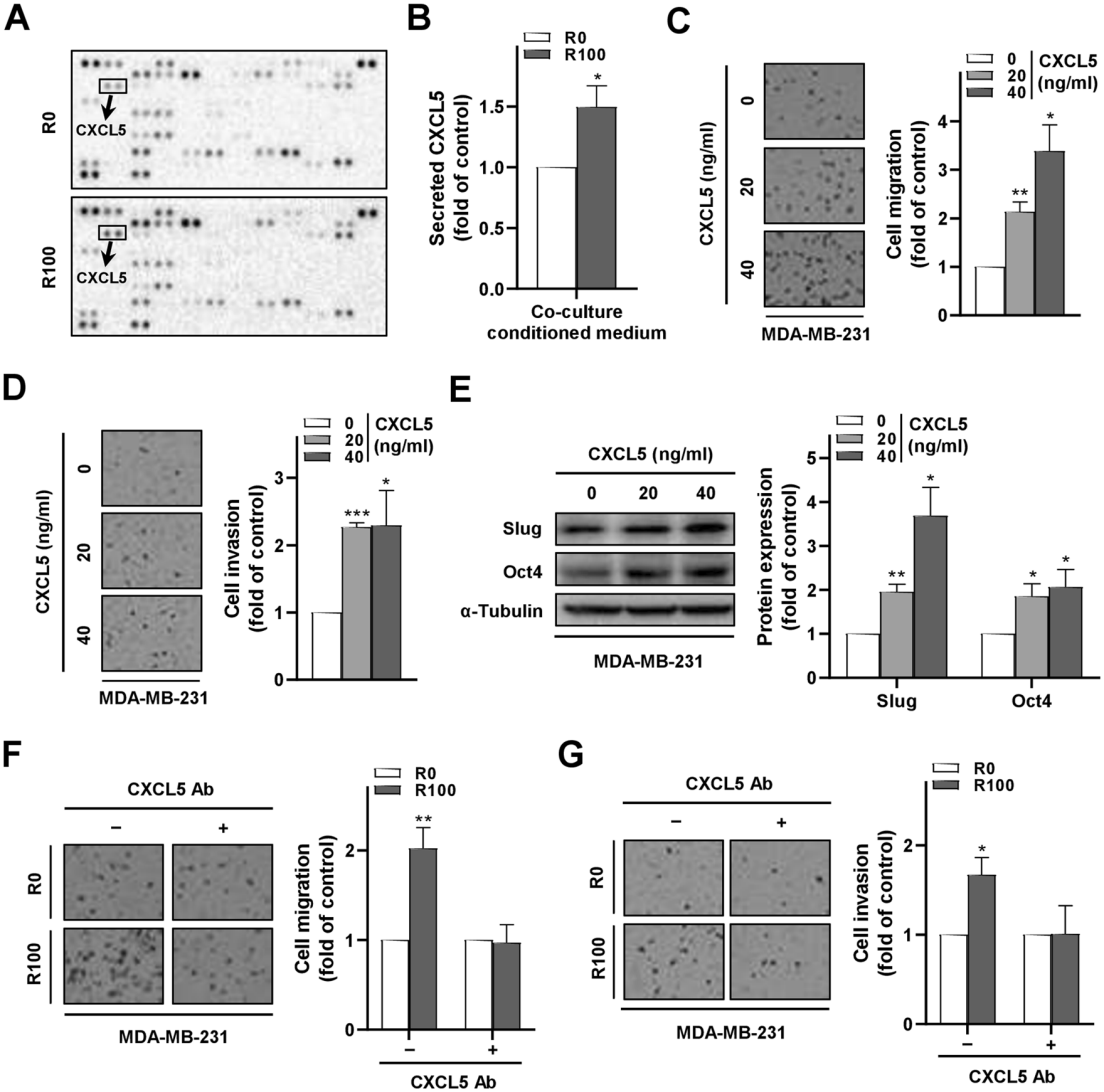
Secreted CXCL5 in the conditioned medium of the co-culture model with resistin-stimulated ADSCs promoted malignant behaviors of breast cancer cells. The isolated ADSCs were treated with resistin at 0 and 100 ng/ml (R0 and R100, respectively) for 48 h, followed by co-culture with MDA-MB-231 cells in the transwell model for another 72 h before the analyses in (A,B,F,G). (**A**) The conditioned medium from the co-culture of R-ADSCs (R100) or control ADSCs (R0) with MDA-MB-231 cells was collected and analyzed by cytokine/chemokine proteome array. A total of 102 proteins were detected with duplicates for each protein. The position of CXCL5 on the array was highlighted. (**B**) Secreted protein level of CXCL5 in the conditioned medium from the co-culture of R-ADSCs (R100) or control ADSCs (R0) with MDA-MB-231 cells was analyzed by ELISA. (**C**) MDA-MB-231 cells treated with recombinant CXCL5 (0, 20, and 40 ng/ml) for 48 h were collected and evaluated by cell migration assay. (**D**) MDA-MB-231 cells treated with recombinant CXCL5 (0, 20, and 40 ng/ml) for 48 h were collected and evaluated by cell invasion assay. (**E**) MDA-MB-231 cells treated with recombinant CXCL5 (0, 20, and 40 ng/ml) for 48 h were collected and analyzed for the protein expression of mesenchymal marker Slug and cancer stemness marker Oct4 by Western blot. The original blot images in (A) and (E) were available in Supplementary Information. (**F,G**) CXCL5 neutralizing antibody was added ( +) or omitted (–) during the co-culture of R-ADSCs (R100) or control ADSCs (R0) with MDA-MB-231 cells. After the co-culture, MDA-MB-231 cells were collected and evaluated by cell migration assay in (F) and cell invasion assay in (G). Data were obtained from three independent experiments and presented as mean ± SEM. Statistical difference was determined by t-test comparing R100 group versus their corresponding R0 group as control, or comparing recombinant CXCL5 treatment group (20 and 40 ng/ml) versus recombinant CXCL5 control group (0 ng/ml). *p < 0.05; **p < 0.01; ***p < 0.001.

To search for the critical mediator involved in R-ADSC-promoted breast cancer cell malignant behaviors, we utilized a proteome array to screen a range of human cytokines and chemokines in the conditioned medium collected from the co-culture model. Notably, the chemokine CXCL5 was found to be the top secreted protein among 102 proteins screened in the conditioned medium of the co-culture group containing R-ADSCs compared with the control group (Fig. 3A and Supplementary Fig. S3). As CXCL5 may have a crucial role in the tumor microenvironment for breast cancer cell growth and metastasis^24–27^, we focused our following investigations on this chemokine to further characterize its involvement in R-ADSC-mediated breast cancer malignant behaviors. The increase of CXCL5 on the array was confirmed by ELISA, which showed an elevated level of CXCL5 in the conditioned medium of MDA-MB-231 co-cultured with R-ADSCs compared with the control (Fig. 3B). In addition, MDA-MB-231 cells treated with recombinant CXCL5 proteins not only showed increased cell migration and invasion ability (Fig. 3C,D), but also showed enhanced expression of the mesenchymal marker Slug and stemness marker Oct4 (Fig. 3E). Furthermore, we applied a CXCL5 neutralizing antibody to antagonize the secreted CXCL5 in the conditioned medium, and found that the presence of CXCL5 neutralizing antibody abolished the migration and invasion ability in MDA-MB-231 cells after co-culture with R-ADSCs (Fig. 3F,G). Together, these data suggest that the induced secretion of CXCL5 in the co-culture system played a critical role in promoting breast cancer cell malignancy.

### Enhanced breast cancer cell migration by resistin-stimulated ADSCs was associated with extracellular signal-regulated kinase (ERK) phosphorylation.

**Figure 4:**
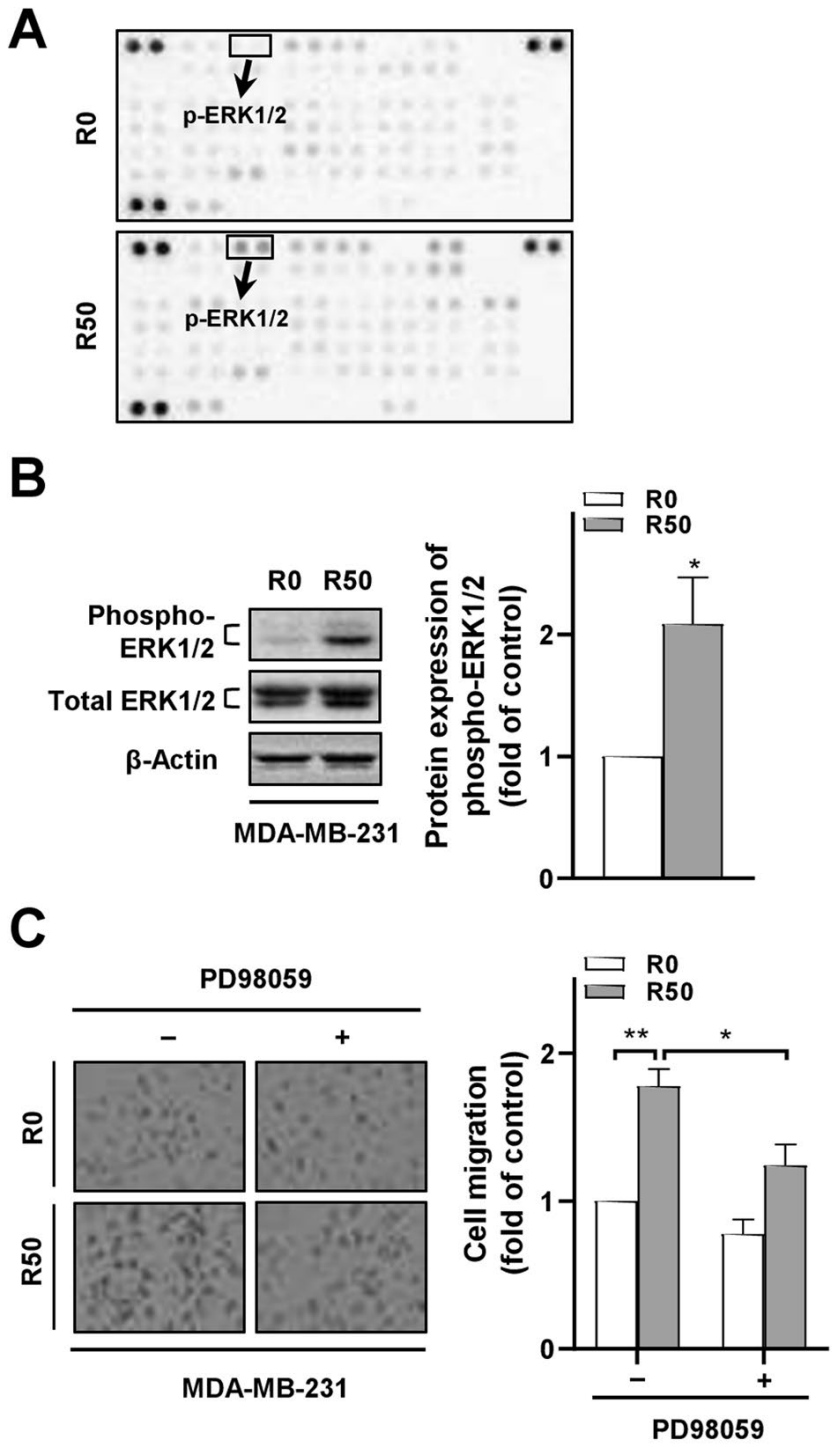
ERK phosphorylation in breast cancer cells after co-culture with resistin-stimulated ADSCs mediated the cancer cell migration ability. The isolated ADSCs were treated with resistin at 0 and 50 ng/ml (denoted as R0 and R50, respectively) for 48 h, followed by co-culture with breast cancer cells in the transwell model for another 72 h before the following analyses. (**A**) Total protein lysates from MDA-MB-231 cells after co-culture with R-ADSCs (R50) or control ADSCs (R0) were collected and analyzed by phospho-kinase proteome array. A total of 43 phosphorylation sites were detected with duplicates for each protein. The position of phospho-ERK1/2 (p-ERK1/2) on the array was highlighted. (**B**) MDA-MB-231 cells after co-culture with R-ADSCs (R50) or control ADSCs (R0) were collected and analyzed for the protein expression of phospho-ERK1/2 and total ERK1/2 by Western blot. The original blot images in (A) and (B) were available in Supplementary Information. (**C**) PD98059 (5 μM) was added (+) or omitted (–) during the co-culture of MDA-MB-231 with R-ADSCs (R50) or control ADSCs (R0). After the co-culture, MDA-MB-231 cells were collected and evaluated by cell migration assay. Data were obtained from three independent experiments and presented as mean ± SEM. Statistical difference was determined by t-test comparing R50 group versus R0 group as control, or comparing R50 group with PD98059 ( +) versus R50 group without PD98059 (–). *p < 0.05; **p < 0.01.

To elucidate signaling molecules associated with breast cancer cell malignancy induced by R-ADSCs, we screened the phosphorylation status of protein kinases in MDA-MB-231 cells from the co-culture model by a phospho-kinase proteome array. As shown in Fig. 4A and Supplementary Fig. S4, MDA-MB-231 cells after co-culture with R-ADSCs revealed multiple changes of protein phosphorylation, with phosphorylated ERK showing the most increased level out of 43 kinase phosphorylation sites screened on the array compared with the control. The elevated phosphorylation of ERK in MDA-MB-231 cells under the same co-culture condition was further confirmed by Western blot (Fig. 4B). Since activation of ERK signaling has been associated with CXCL5-promoted malignancy in breast cancer^26^, we applied PD98059, a potent inhibitor for ERK phosphorylation^35^, to investigate its influence on breast cancer cell migration in the co-culture model. The results showed that application of PD98059 largely reduced the migration ability of MDA-MB-231 cells after co-culture with R-ADSCs compared with the group without PD98059 treatment (Fig. 4C), suggesting the participation of ERK pathway in breast cancer cell malignancy induced by resistin-stimulated ADSCs.

### Resistin-stimulated ADSCs enhanced breast tumor growth in xenograft mice with upregulated expression of CXCL5, Slug, and ERK phosphorylation

**Figure 5:**
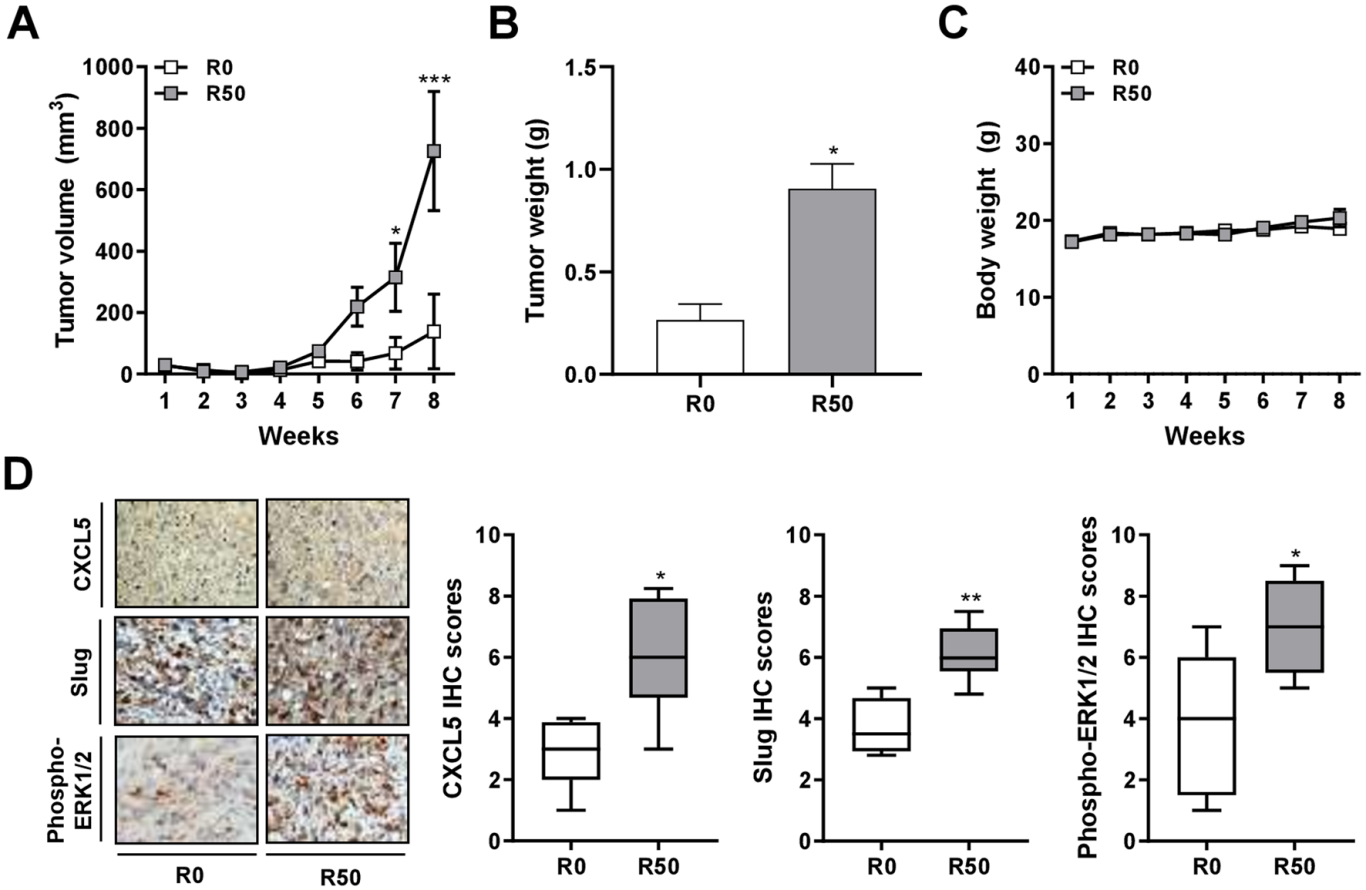
Enhanced breast tumor growth and protein expression of CXCL5, Slug, and ERK phosphorylation in mice via xenograft of breast cancer cells after co-culture with resistin-stimulated ADSCs. The isolated ADSCs were treated with resistin at 0 and 50 ng/ml (denoted as R0 and R50, respectively) for 48 h, followed by co-culture with MDA-MB-231 cells in the transwell model for another 72 h. After the co-culture, MDA-MB-231 cells were collected and injected into the fourth mammary fat pads of female NOD/SCID mice for the following analyses. (**A**) The tumor volume, calculated by (width^2^ × length)/2, was measured weekly after palpable tumor mass was formed. (**B**) Upon sacrifice of the mice on week eight, the weight of individual tumor mass was measured. (**C**) The body weight of the mice was measured weekly. (**D**) The tumor mass was collected after sacrifice of the mice on week eight, and analyzed for CXCL5, Slug, and phospho-ERK1/2 protein expression by immunohistochemistry (IHC). The quantitative IHC scores were manually evaluated and calculated by multiplying the categorized percentage of stained cells (0, 0–24%; 1, 25‑49%; 2, 50‑74%; 3, 75‑100%) by the categorized intensity of staining (0, negative; 1, weak; 2, moderate; 3, strong). Data were obtained from three to six mice in each group and presented as mean ± SEM or box plots. Statistical difference was determined by t-test comparing R50 group versus R0 group as control. *p < 0.05; **p < 0.01; ***p < 0.001.

To explore the effect of resistin-stimulated ADSCs on breast tumor development in vivo, we collected the MDA-MB-231 cells after co-culture with R-ADSCs, or control ADSCs without resistin treatment, for xenograft implantation in the mammary fat pads of NOD/SCID mice. The tumor volume at these orthotopic sites was measured weekly after palpable tumor mass was formed, which began to show a significant difference between the experimental and control groups on week seven (Fig. 5A). The tumor weight was measured at the end point of the experiment on week eight after collection of the xenograft tumors, confirming that the tumor growth from the group of MDA-MB-231 co-cultured with R-ADSCs was significantly increased compared with the control group (Fig. 5B). The body weight of these mice was not significantly different between the two groups within the eight-week observation period (Fig. 5C). The collected xenograft tumors were analyzed by immunohistochemical analysis, which revealed an elevated expression level of CXCL5, Slug, or ERK phosphorylation in the group of MDA-MB-231 co-cultured with R-ADSCs compared with the control group (Fig. 5D). These data suggest that the xenograft breast tumor growth enhanced by resistin-stimulated ADSCs was potentially associated with the increased expression of CXCL5 and mesenchymal markers, such as Slug, along with the activation of ERK pathway.

### Correlation of resistin, CXCL5, and phosphorylated ERK expression in breast cancer patients

**Figure 6:**
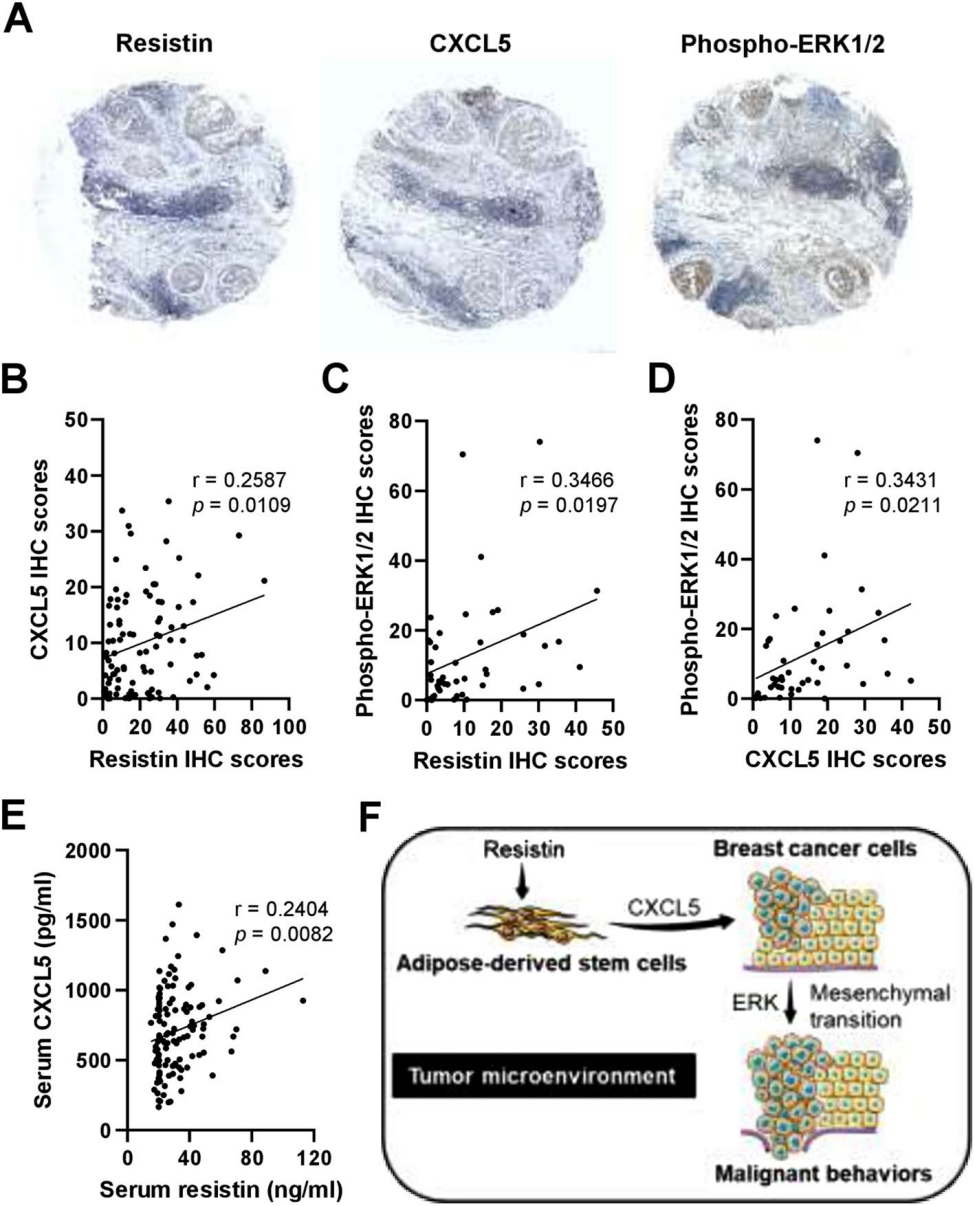
Correlation analysis for the protein expression of resistin, CXCL5, and ERK phosphorylation in the tumor and serum specimens from breast cancer patients. (**A**) Representative images of immunohistochemistry (IHC) staining for resistin, CXCL5, and phospho-ERK1/2 expression in breast tumor sections from breast cancer patients. (**B–D**) The quantitative IHC scores were evaluated by HistoQuest software, followed by Pearson correlation (r) analysis between resistin and CXCL5 in (**B**) (n = 96), resistin and phospho-ERK1/2 in (**C**) (n = 45), and CXCL5 and phospho-ERK1/2 in (**D**) (n = 45). (**E**) The serum levels of resistin and CXCL5 in breast cancer patients were determined by ELISA, followed by Pearson correlation (r) analysis (n = 120). The mean ± SD of resistin and CXCL5 was 31.4 ± 15.4 ng/ml and 707.9 ± 293.4 pg/ml, respectively. (**F**) Schematic summary of the current study. Our preclinical and clinical data together suggest that CXCL5 may be secreted by resistin-stimulated ADSCs in the breast tumor microenvironment, promoting breast cancer cell malignancy via the participation of ERK pathway and epithelial-to-mesenchymal transition. The schematic summary in (**F**) was produced using the illustration elements from Servier Medical Art (https://smart.servier.com), which is in compliance with the terms of the Creative Commons Attribution 3.0 Unported License (https://creativecommons.org/licenses/by/3.0/).

Based on the results of our preclinical investigation, we further analyzed whether clinical correlations occur between the expression levels of resistin, CXCL5, and ERK phosphorylation in breast cancer patients by immunohistochemistry (Fig. 6A). Notably, resistin expression was found to positively correlate with CXCL5 expression in the breast tumor tissues of the patients (Fig. 6B). In addition, a positive correlation was observed between resistin and phosphorylated ERK expression (Fig. 6C), as well as that between CXCL5 and phosphorylated ERK expression (Fig. 6D). We also examined the serum levels of resistin and CXCL5 in breast cancer patients by ELISA, and the results showed a positive correlation between the two proteins (Fig. 6E). These clinical data and our preclinical findings together suggest the potential of CXCL5 secretion induced by resistin-stimulated ADSCs in the breast tumor microenvironment, and that promoted breast cancer cell malignancy with the participation of ERK signaling pathway (Fig. 6F).

## Discussion

The role of the tumor microenvironment in cancer management is increasingly important, with the attention of stromal cell-initiated tumor growth and metastasis highlighted in breast cancer and other cancer types^36–38^, yet the underlying mechanisms of interaction between cancer and stromal cells remain to be elucidated. In this study, the potential interaction between ADSCs and breast cancer cells was investigated, implicating CXCL5, a breast cancer-associated chemokine^24–30^, to be a mediator that promotes malignant behaviors of breast cancer cells under the paradigm of resistin-stimulated ADSCs. As summarized in Fig. 6F, the cancer promoting effect via the proposed route of resistin-ADSC-CXCL5 may involve activation of the ERK pathway and epithelial-to-mesenchymal transition in breast cancer cells. Our clinical analyses further support this view, with evidence to show a mutual correlation between the expression of resistin, CXCL5, and ERK phosphorylation in the tumor tissues of breast cancer patients.

Elevated CXCL5 expression in breast cancer has been associated with adverse clinical outcomes, such as increased primary tumor size^29^, high grade metastasis^28^, and poor metastasis-free survival^30^. In addition, CXCL5 secreted by healthy donor-derived ADSCs showed an enhancing effect on breast cancer cell proliferation^27^. In the present study, we further considered the activation state of patient-derived ADSCs stimulated by resistin, an adipocytokine associated with adverse breast cancer progression^12–15^, and their impact on breast cancer cell malignancy in the tumor microenvironment. The breast tumor microenvironment has been known as a network where crosstalk occurs between breast cancer cells and diverse stromal components, making extrapolation of their interactions complicated^16,22^. Therefore, the results of our present study by dissecting the interaction between ADSCs and breast cancer cells may provide valued information, suggesting an upstream regulation of CXCL5 secretion from ADSCs induced by resistin, which has not been defined before, to promote breast cancer cell malignancy in the context of the tumor microenvironment.

The secretory resistin in human has been found to be released from adipose tissues, with a larger portion from non-adipocytic stromal cells and a smaller portion from adipocytes^8–10^. Nevertheless, the biological mechanisms of resistin occurring in the breast tumor microenvironment that contains abundant adipose tissues remain largely unidentified, although elevated resistin expression has been observed in breast tumor and serum samples of breast cancer patients^12–15^. In this study, a novel pathway of resistin-promoted breast cancer was highlighted with the effect of resistin indirectly promoting breast cancer cell malignancy via CXCL5 secretion in the tumor microenvironment, leading to the downstream ERK activation and epithelial-to-mesenchymal transition towards mesenchymal phenotypes in breast cancer cells. This indirect mechanism is noticeably distinct from the direct effect of resistin on breast cancer cells, such as the enhanced tumor growth and metastatic ability of breast cancer cells via toll-like receptor 4 (TLR4) or adenylyl cyclase-associated protein 1 (CAP1)-mediated pathways^15,39^. These direct and indirect effects of resistin may work in synergy in the breast tumor microenvironment, and therefore targeting the direct pathway of resistin, or its indirect pathway, such as CXCL5 reported in this study, may be equally important for future development of therapeutic intervention in the breast cancer associated with elevated expression of resistin.

Our current study provides a novel link between resistin and CXCL5 under the consideration of cell–cell interaction in the tumor microenvironment. However, there are also limitations in the interpretation of our current data, and areas that merit further investigation. For example, whether our current findings from co-culture of ADSCs with MCF-7 (luminal type A) and MDA-MB-231 (basal type) occur in other molecular surrogates of breast cancer cells, such as luminal type B, HER2-overexpressing, or non-malignant mammary epithelial cells^40^, remain to be determined. There is also possibility that CXCL5 may be co-released by breast cancer cells as an autocrine or paracrine factor to promote malignancy in our transwell co-culture model. In addition, while we have conducted in vivo investigation for orthotopic primary tumor growth in this study, the impact of resistin-stimulated ADSCs on the outcome of distant metastasis, in particular the bones, lungs, and livers as the most common sites in breast cancer^41^, remains to be explored. Furthermore, future studies for protein expression on breast tumors by immunohistochemistry may consider developing double-staining for the expression of CXCL5 and ADSC markers, such as CD44, CD90, and CD105^31,32^, or the expression of CXCL5 and tumor markers for fast replicating cancer cells, such as Ki67 and proliferating cell nuclear antigen (PCNA)^42,43^, to differentiate the expression loci of CXCL5 in the breast tumor microenvironment. Another consideration is that the pleiotropic effect of resistin is not restricted to cancer-associated ADSCs, thus its influence on ADSCs harbored in non-cancerous mammary tissues^44^ or non-mammary adipose tissues^45^, as well as other responsive stromal constituents, such as cancer-associated fibroblasts or macrophages^16^, merit further investigation. Recent advance of single-cell multi-omics may provide a precision medicine-oriented approach in the investigation of these complex cell–cell interactions^46^.

## Conclusions

The current study adds to the growing body of evidence indicating that tumor-stroma interactions are of significant importance in breast cancer, and specifically these data indicate that resistin may act via mechanistically distinct pathways from those previously discovered using tumor cell models alone in isolation. These newly discovered pathways of resistin may be mediated through ADSCs in the breast tumor microenvironment via CXCL5 secretion, leading to the malignant behaviors of breast cancer cells. This may have implications not only on that the resistin-ADSC-CXCL5 pathway as a therapeutic target, but on the re-evaluation of factors that may affect ADSC activities in the tumor microenvironment, such as neoadjuvant and adjuvant therapies, and post-surgical autologous fat grafting in breast cancer patients^47^. Furthermore, the co-culture model of ADSCs with breast cancer cell lines reported in this study may be applied to investigate the impact of ADSCs on patient-derived tumor xenografts (PDXs) to recapitulate the complexity of the breast tumor microenvironment^48^.

## Methods

### Human specimens

Ninety-six breast tumor specimens, and one hundred and twenty serum samples, were collected from female patients who were diagnosed with invasive breast ductal carcinoma and underwent surgical treatment at Kaohsiung Medical University Hospital (KMUH; Kaohsiung, Taiwan). Ten adipose tissue specimens adjacent to breast tumors were randomly collected from the patients during surgical treatment and used in this study. The clinical characteristics of ADSC donors were summarized in Supplementary Table S1. This study was approved by the Institutional Review Board of Kaohsiung Medical University Hospital (approval no. KMUH-IRB-20130347), and informed consent was obtained from all participants.

### Cell culture

Human breast cancer cell lines MDA-MB-231 (RRID: CVCL_0062) and MCF-7 (RRID: CVCL_0031) were obtained from the Bioresource Collection and Research Center (BCRC; Hsinchu, Taiwan) and cultured in Dulbecco's Modified Eagle Medium (DMEM; Thermo Fisher Scientific, Waltham, MA, USA) containing 10% fetal bovine serum (FBS; Biological Industries, Beit Haemek, Israel) and 1% penicillin–streptomycin-amphotericin B solution (Thermo Fisher Scientific, Waltham, MA, USA). These human cell lines used for this study were authenticated by short tandem repeats (STR) profiling (Topgen Biotechnology; Kaohsiung, Taiwan), and routinely tested for mycoplasma by polymerase chain reaction^49^. The procedure for ADSC isolation from human adipose tissues was reported previously^50^. Briefly, adipose tissues adjacent to the breast tumor region were collected during surgical treatment of the patients. These adipose tissues were cut into small pieces, followed by digestion of the extracellular matrix with 1.5 mg/ml collagenase type II (Sigma-Aldrich, St. Louis, MO, USA) in 1% bovine serum albumin dissolved in phosphate-buffered saline (PBS; Sigma-Aldrich, St. Louis, MO, USA) for 1 h at 37℃ on a rotor. The digested tissue solution was passed across 100 μm cell strainers (Corning, Tewksbury, MA, USA) and centrifuged at 500 g for 10 min. The cell pellet was washed with PBS and cultured in Minimum Essential Medium Alpha Modification (α-MEM; Hyclone, Logan, UT, USA) containing 5% UltraGRO (AventaCell, Atlanta, GA, USA) and 1% penicillin–streptomycin-amphotericin B solution (Thermo Fisher Scientific, Waltham, MA, USA). All cells were cultured under 5% CO_2_ atmosphere in a humidified 37℃ incubator.

### Flow cytometry

After culturing for three passages, the ADSCs were collected and re-suspended in sorting buffer (1% FBS dissolved in PBS) for detection of cell surface markers. Fluorochrome-conjugated mouse monoclonal antibodies against human CD34 (clone 4H11), CD44 (clone IM7), CD45 (clone HI30), CD90 (clone 5E10), or CD105 (clone SN6) from Thermo Fisher Scientific (Waltham, MA, USA) were incubated with the ADSCs for 30 min on ice. After the incubation, the cells were washed with PBS and re-suspended in the sorting buffer before analysis by Cytomics FC 500 Flow Cytometer equipped with CXP software ver. 2.2 (Beckman Coulter, Brea, CA, USA).

### ADSC differentiation assays

The multipotency of isolated ADSCs was tested by lineage-specific differentiation following the previously established methods^50^. Briefly, adipogenesis was induced by culturing ADSCs for 12 days in an adipogenic medium, followed by evaluation for lipid accumulation with Oil Red O stain (Sigma-Aldrich, St. Louis, MO, USA). Osteogenesis was induced by culturing ADSCs in an osteogenic medium for 2 weeks, followed by evaluation for calcium deposits with silver plating-von Kossa stain (Sigma-Aldrich, St. Louis, MO, USA). Chondrogenesis was induced by culturing ADSCs in a chondrogenic medium for 2 weeks, followed by evaluation for extracellular sulfated/phosphated proteoglycan-rich matrices with Alcian Blue 8GX stain (Sigma-Aldrich, St. Louis, MO, USA). The complete cell culture medium for each lineage-specific differentiation was prepared according to the previous report^50^.

### Transwell co-culture model

The ADSCs were plated in 6-well plates (2 × 10^5^/well) overnight, followed by serum starvation for 24 h. These ADSCs were then treated with resistin (denoted as R-ADSCs) (PeproTech, Rehovot, Israel) at 50 or 100 ng/ml for 48 h, and those without resistin treatment served as control ADSCs. After the treatment, the ADSCs were re-plated in the inserts (2 × 10^4^ cells/insert; 0.4 µm pores) of the 24-well transwell plates (Corning, Tewksbury, MA, USA), and MDA-MB-231 or MCF-7 cells were plated in the bottom wells (6 × 10^4^ cells/well). In some experimental conditions, CXCL5 neutralizing antibody (5 μg/ml; R&D Systems, Minneapolis, MN, USA) or PD98059 (5 μM; Sigma-Aldrich, St. Louis, MO, USA) were added to the bottom wells. After co-culture for 72 h, the conditioned medium, or the cells in the bottom wells, were collected for further analysis.

### Cell viability assay

MDA-MB-231 cells after co-culture with R-ADSCs or control ADSCs were collected and plated in 96-well plates (2 × 10^3^ cells/well), and cultured for another 24, 48, or 72 h. XTT assay for viable cells was performed by replacing the cell culture medium with XTT solution (Sigma-Aldrich, St. Louis, MO, USA) at each time point, and viability readouts were calculated by subtraction of the main absorbance wavelength at 475 nm from reference absorbance wavelength at 660 nm.

### Cell migration and invasion assays

MDA-MB-231 or MCF-7 cells after co-culture with R-ADSCs or control ADSCs were collected and re-suspended in serum-free DMEM, and plated in the inserts (2 × 10^4^ cells/insert; 8 μm pores) of the 24-well transwell plates (Corning, Tewksbury, MA, USA). The inserts were either with or without Matrigel coating (Corning, Tewksbury, MA, USA) for cell invasion or migration assay, respectively. The bottom wells were supplied with DMEM containing 10% FBS. After 24 h, cells remaining on the upper side of the insert membrane were removed by cotton swabs, while cells appearing on the underside of the insert membrane were fixed with 4% formaldehyde (Sigma-Aldrich, St. Louis, MO, USA) and stained with 0.05% crystal violet (Sigma-Aldrich, St. Louis, MO, USA). Images of the crystal violet staining were captured under a light microscope and analyzed by ImageJ software (https://imagej.nih.gov/ij/).

### Soft agar colony formation assay

A supporting base layer of soft agar in 6-well plates was prepared by dissolving 0.5% low-melting agarose (Sigma-Aldrich, St. Louis, MO, USA) in DMEM containing 10% FBS. The cell-containing layer was further prepared as follows: MDA-MB-231 cells after co-culture with R-ADSCs or control ADSCs were collected and re-suspended in DMEM containing 10% FBS mixed with 0.35% low-melting agarose, followed by plating the mixture (5 × 10^3^ cells/well) on top of the base soft agarose layer in 6-well plates. After culturing for 14 days, the colonies were stained with 0.25% crystal violet, and images of the crystal violet staining were captured under a dissection microscope and analyzed by ImageJ software.

### Tumorsphere formation assay

MDA-MB-231 or MCF-7 cells after co-culture with R-ADSCs or control ADSCs were collected and re-suspended in phenol red-free DMEM (Thermo Fisher Scientific, Waltham, MA, USA) containing 20 ng/ml recombinant human epidermal growth factor (PeproTech, Rehovot, Israel), 20 ng/ml recombinant human fibroblast growth factor-basic (PeproTech, Rehovot, Israel), 1 × B27 (Thermo Fisher Scientific, Waltham, MA, USA), and 10 μg/ml insulin (Thermo Fisher Scientific, Waltham, MA, USA). These cells were transferred to the ultra-low attachment 96-well plates (2 × 10^3^ cells/well; Corning, Tewksbury, MA, USA) and cultured for 7 days prior to assess the formation of tumorspheres, which were imaged under a light microscope and analyzed by ImageJ software.

### Western blot

MDA-MB-231 cells after co-culture with R-ADSCs or control ADSCs were collected, and their total protein lysates were extracted with RIPA buffer (Sigma-Aldrich, St. Louis, MO, USA). Equal amount of the extracted proteins from each sample was loaded onto SDS-PAGE with a Mini-PROTEAN electrophoresis system (Bio-Rad, Hercules, CA, USA), and transferred to PVDF membranes (Merck Millipore) with a Mini Trans-Blot system (Bio-Rad, Hercules, CA, USA). The membranes were blocked with 5% non-fat milk, followed by incubation with designated primary antibodies at 4℃ overnight. After washing with TBST (Tris-buffered saline containing 0.1% Tween-20; Sigma-Aldrich, St. Louis, MO, USA), the membranes were incubated with species-matched horseradish peroxidase-conjugated secondary antibodies (Thermo Fisher Scientific, Waltham, MA, USA) for 1 h at room temperature. Immunoreactive proteins were detected by immersing the membranes with chemiluminescent reagents (Merck Millipore, Burlington, MA, USA), and the images were acquired by ChemiDoc XRS + imaging system (Bio-Rad, Hercules, CA, USA). Quantification for protein expression was performed by Image Lab software (Bio-Rad, Hercules, CA, USA). The primary antibodies used in this study for Western blot are listed as follows: rabbit polyclonal antibodies against human β-actin, α-tubulin, Oct4, Snail, Slug, Vimentin, and Nanog were from GeneTex (Hsinchu, Taiwan); mouse monoclonal antibody against human ERK1/2 (clone E31R) was from GeneTex (Hsinchu, Taiwan); rabbit monoclonal antibody against human phosphorylated ERK1/2 at Thr202/Tyr204 (clone D13.14.4E) was from Cell Signaling Technology (Danvers, MA, USA).

### Proteome arrays

To explore protein profiles of released cytokines/chemokines in the co-culture model, the conditioned medium from the co-culture of MDA-MB-231 with R-ADSCs or control ADSCs was collected and analyzed by the Human XL Cytokine Array kit (R&D Systems, Minneapolis, MN, USA). To explore signaling pathways involved in breast cancer cell malignancy in the co-culture model, total protein extracts from MDA-MB-231 cells after co-culture with R-ADSCs or control ADSCs were analyzed by the Human Phospho-Kinase Array kit (R&D Systems, Minneapolis, MN, USA). The procedures for both arrays were carried out according to the manufacturer’s instructions.

### Enzyme-linked immunosorbent assay (ELISA)

To confirm the secretion of CXCL5 in the co-culture model, the conditioned medium from the co-culture of MDA-MB-231 with R-ADSCs or control ADSCs was collected and analyzed by the Human CXCL5/ENA-78 DuoSet ELISA kit (R&D Systems, Minneapolis, MN, USA). The serum samples of breast cancer patients were analyzed by the Human Resistin Quantikine ELISA kit (R&D Systems, Minneapolis, MN, USA) for resistin expression, and by the Human CXCL5/ENA-78 DuoSet ELISA kit for CXCL5 expression. The procedures for these ELISA kits were carried out according to the manufacturer's instructions.

### Animal study

All experiments on animals were approved by the Institutional Animal Care and Utilization Committee of Kaohsiung Medical University (Kaohsiung, Taiwan), and conducted in accordance with ARRIVE guidelines (https://arriveguidelines.org). After co-culture with R-ADSCs or control ADSCs, the MDA-MB-231 cells were collected for orthotopic xenograft experiments in mice. These MDA-MB-231 cells were re-suspended in normal saline (2 × 10^6^ cells), followed by injection into the fourth mammary fat pads of 6-week-old female non-obese diabetic/severe combined immunodeficiency (NOD/SCID) mice. The tumor size was measured weekly by the formula of (width^2^ × length)/2 after the tumor mass was palpable (approximately 4 weeks post-xenograft). At the end point of experiments, all mice were euthanized and sacrificed for tumor weight measurement and immunohistochemical analysis.

### Immunohistochemistry (IHC)

Paraffin-embedded and formalin-fixed tumor sections from breast cancer patients and orthotropic xenograft mice were immunostained by the Bond-Max automated IHC stainer (Leica Microsystems, Wetzlar, Germany) according to the manufacturer’s instructions. The primary antibodies used in this study for IHC are listed as follows: rabbit polyclonal antibody against human Slug (GeneTex, Hsinchu, Taiwan); goat polyclonal antibody against human CXCL5 (R&D Systems, Minneapolis, MN, USA); mouse monoclonal antibody against human resistin (clone C-10; Santa Cruz Biotechnology, Dallas, TX, USA); rabbit monoclonal antibody against human phosphorylated ERK1/2 at Thr202/Tyr204 (clone D13.14.4E; Cell Signaling Technology, Danvers, MA, USA). The images of IHC staining were captured by the TissueFAXS platform (TissueGnostics, Vienna, Austria). For quantitation of IHC staining in the tumor sections from xenograft mice, categorized scores were evaluated blindly and independently by two trained experts and calculated as the product of percentage of stained cells (0, 0–24%; 1, 25–49%; 2, 50–74%; 3, 75–100%) and intensity of staining (0, negative; 1, weak; 2, moderate; 3, strong). In the case of evaluating tumor sections from breast cancer patients, the quantitation of IHC staining was scored by HistoQuest software (TissueGnostics, Vienna, Austria) and further confirmed by a pathologist.

### Statistical analysis

All statistical analyses were performed by GraphPad Prism 8 software (GraphPad Software, San Diego, CA, USA), and data were presented as mean ± SEM where applicable. Student’s t-test was used for comparison between experimental and control groups. Pearson correlation (r) was used to evaluate the correlation of protein expression in human specimens by IHC or ELISA. The results were considered statistically significant if *p* < 0.05.

### Statement

The experiments involving human participants and animals were conducted in accordance with local institutional guidelines and regulations, as well as the Declaration of Helsinki for human research and the ARRIVE guidelines for animal research.

## Supplementary Information

Supplementary Information 1.

Supplementary Information 2.

Supplementary Information 3.

## Data Availability

The data presented in this study are available from the corresponding author on reasonable request.
