# Supplementary material for: ADSCs stimulated by resistin promote breast cancer cell malignancy via CXCL5 in a breast cancer coculture model
Source: Sci Rep. 2022 Sep 14;12:15437. doi: 10.1038/s41598-022-19290-6 (PMC9475041; doi:10.1038/s41598-022-19290-6)
Supplement: Supplementary file 1 — Supplementary Information 1. [file 41598_2022_19290_MOESM1_ESM.pdf]

Supplementary Figure S1

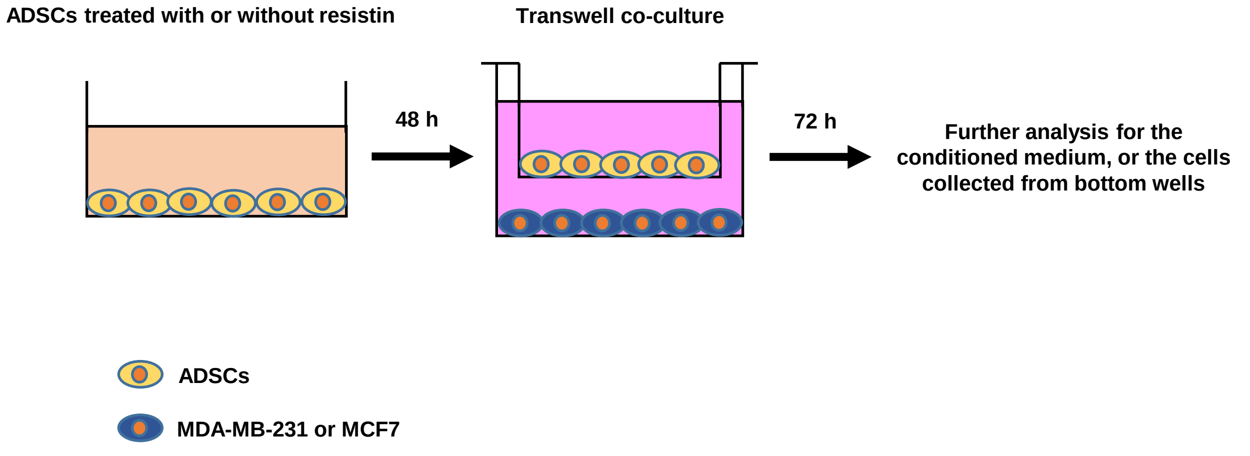

Supplementary Figure S1. Schematic representation of the co-culture model utilized in this study. ADSCs treated with or without resistin for 48 h were re-plated in the inserts of the transwell plates, followed by co-culture with breast cancer cells (MDA-MB-231 or MCF-7) plated in the bottom wells for another 72 h. After the co-culture, the conditioned medium or the cells in the bottom wells were collected and further analyzed by the designated assays as described in the text.

Supplementary Figure S2

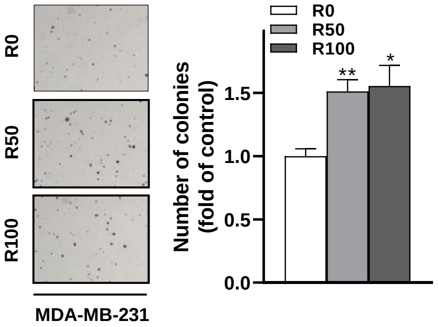

Supplementary Figure S2. Enhanced colony formation of breast cancer cells after co-culture with resistin-stimulated ADSCs. The isolated ADSCs were treated with resistin at 0, 50, and 100 ng/ml (denoted as R0, R50, and R100, respectively) for 48 h, followed by co-culture with MDA-MB-231 cells in the transwell model for another 72 h. After the co-culture, MDA-MB-231 cells were collected and evaluated by soft agar colony formation assay. Quantitation was carried out for those with diameter over 1 mm. Data were obtained from three independent experiments and presented as mean ± SEM. Statistical difference was determined by t-test comparing R50 or R100 group versus R0 group as control. *$p < 0.05$; **$p < 0.01$.

Supplementary Figure S3

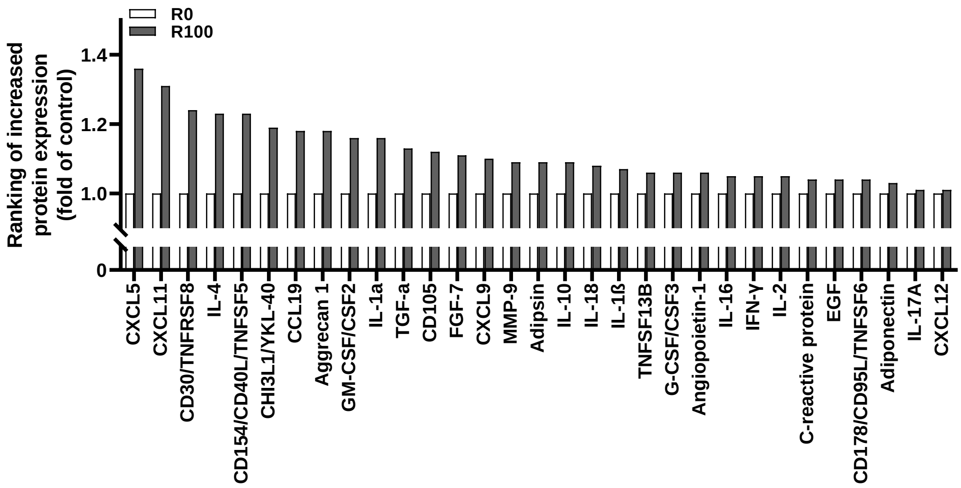

Supplementary Figure S3. Screening for secretory factors in the conditioned medium from the co-culture of resistin-treated ADSCs and breast cancer cells. The isolated ADSCs were treated with resistin at 0 and 100 ng/ml (denoted as R0 and R100, respectively) for 48 h, followed by co-culture with MDA-MB-231 cells in the transwell model for another 72 h. After the co-culture, the conditioned medium was collected and analyzed by cytokine-chemokine proteome array. Each protein expression level on the array was quantified and averaged from duplicates. The increases of protein expression, detected in the conditioned medium of the co-culture with R100 group relative to their corresponding R0 group as control, were presented by rank order. Those with relatively unchanged or decreased values in protein expression were omitted from presentation.

Supplementary Figure S4

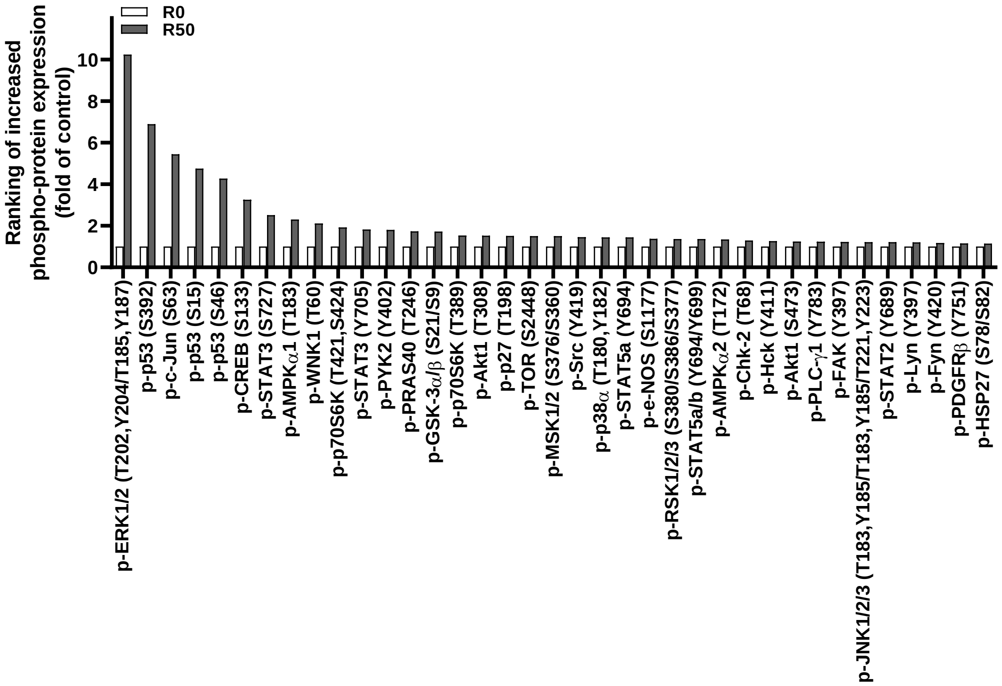

Supplementary Figure S4. Screening for kinase phosphorylation in breast cancer cells co-cultured with resistin-treated ADSCs. The isolated ADSCs were treated with resistin at 0 and 50 ng/ml (denoted as R0 and R50, respectively) for 48 h, followed by co-culture with MDA-MB-231 cells in the transwell model for another 72 h. After the co-culture, total protein lysates of MDA-MB-231 cells were collected and analyzed by phospho-kinase proteome array. The expression level of each phosphorylated protein (denoted with a prefix of “p-“) on the array was quantified and averaged from duplicates. The increases of phospho-protein expression, detected in MDA-MB-231 cells after co-culture with R50 group relative to their corresponding R0 group as control, were presented by rank order. Those with relatively unchanged or decreased values in phospho-protein expression were omitted from presentation.
