# Supplementary material for: ADSCs stimulated by resistin promote breast cancer cell malignancy via CXCL5 in a breast cancer coculture model
Source: Sci Rep. 2022 Sep 14;12:15437. doi: 10.1038/s41598-022-19290-6 (PMC9475041; doi:10.1038/s41598-022-19290-6)
Supplement: Supplementary file 2 — Supplementary Information 2. [file 41598_2022_19290_MOESM2_ESM.pdf]

Supplementary materials – Original blot images

Figure 2E:

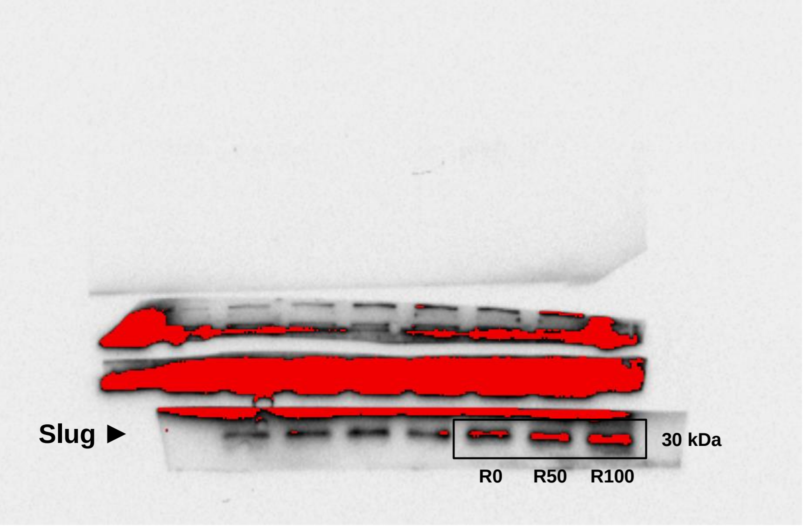

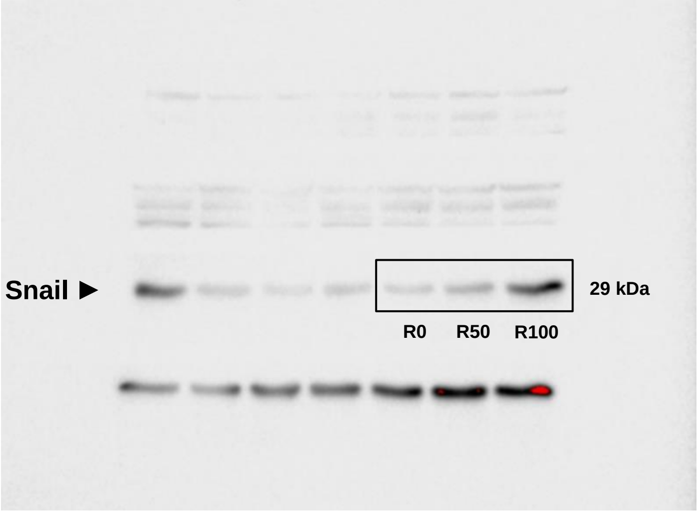

Figure 2E (continued):

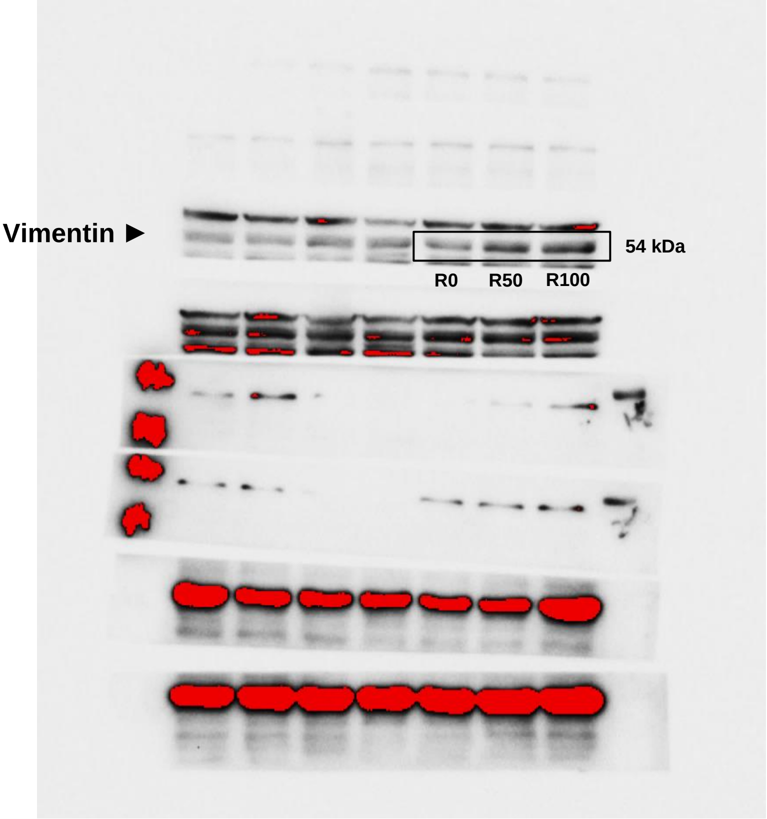

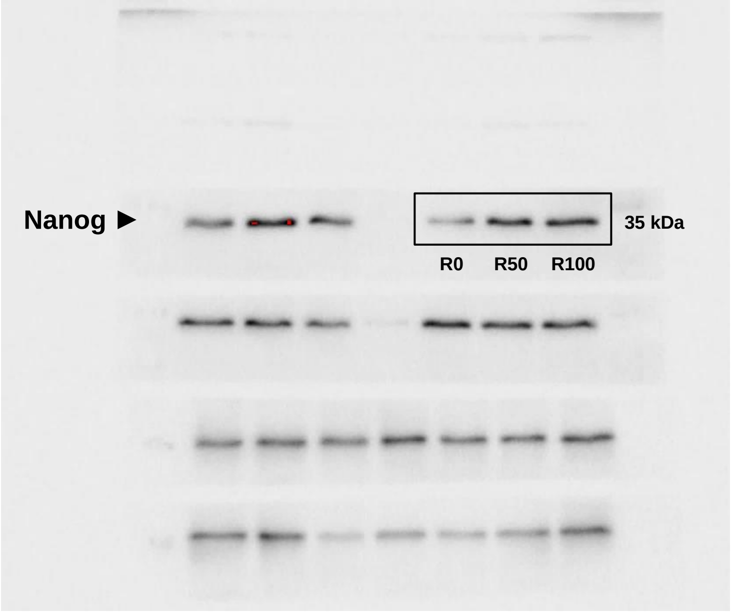

Figure 2E (continued):

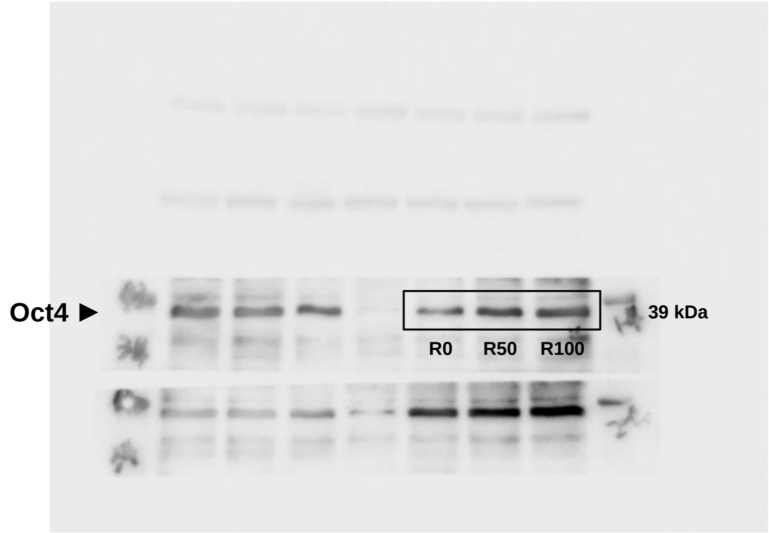

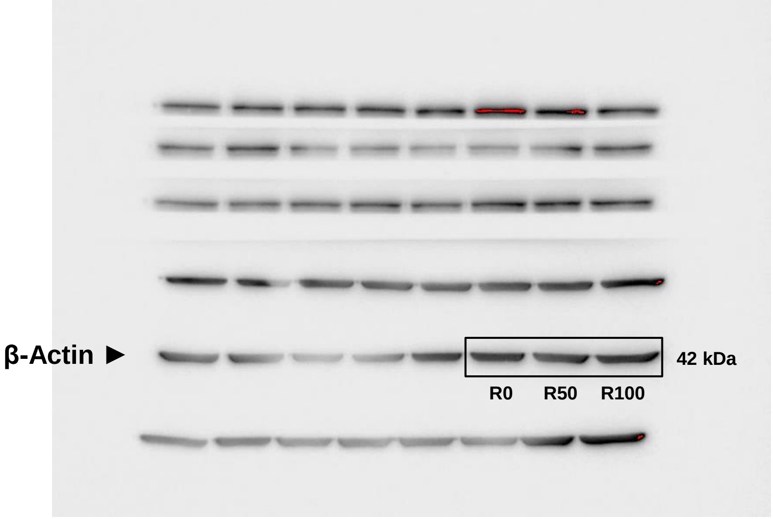

Figure 3A:

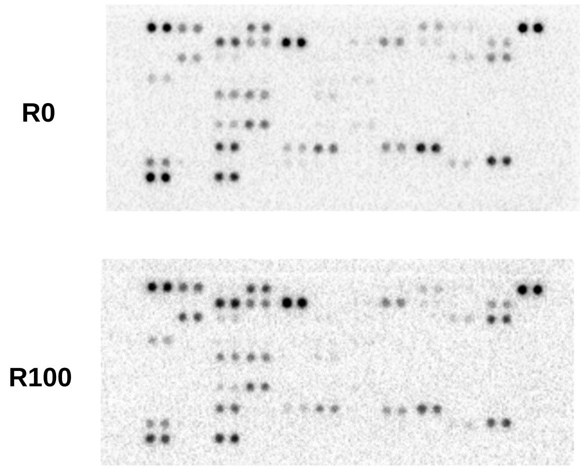

Figure 3E:

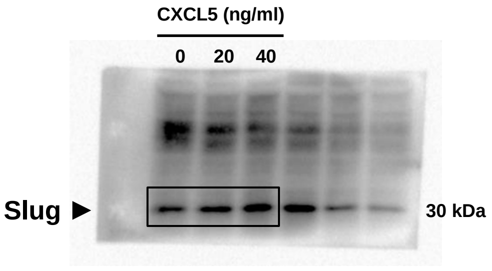
CXCL5 (ng/ml)
0 20 40
Slug
30 kDa

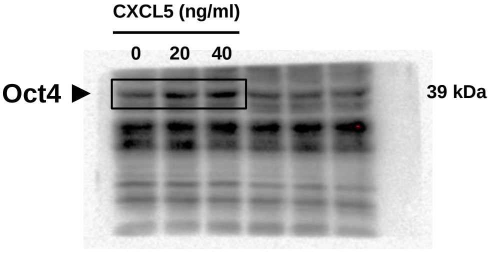
CXCL5 (ng/ml)
0 20 40
Oct4
39 kDa

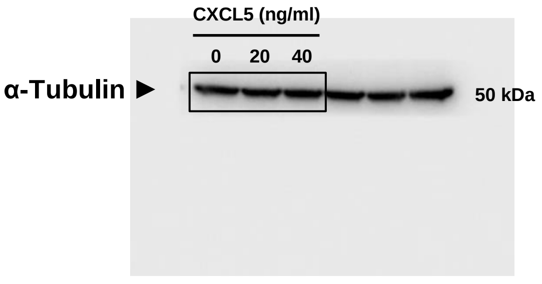
CXCL5 (ng/ml)
0 20 40
α-Tubulin
50 kDa

Figure 4A:

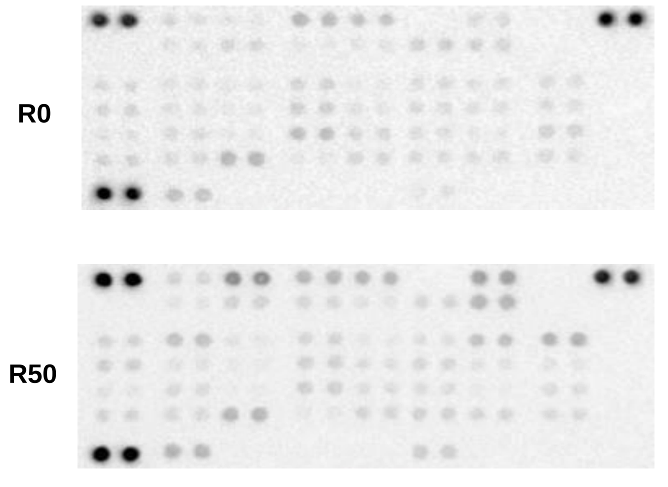

Figure 4B:

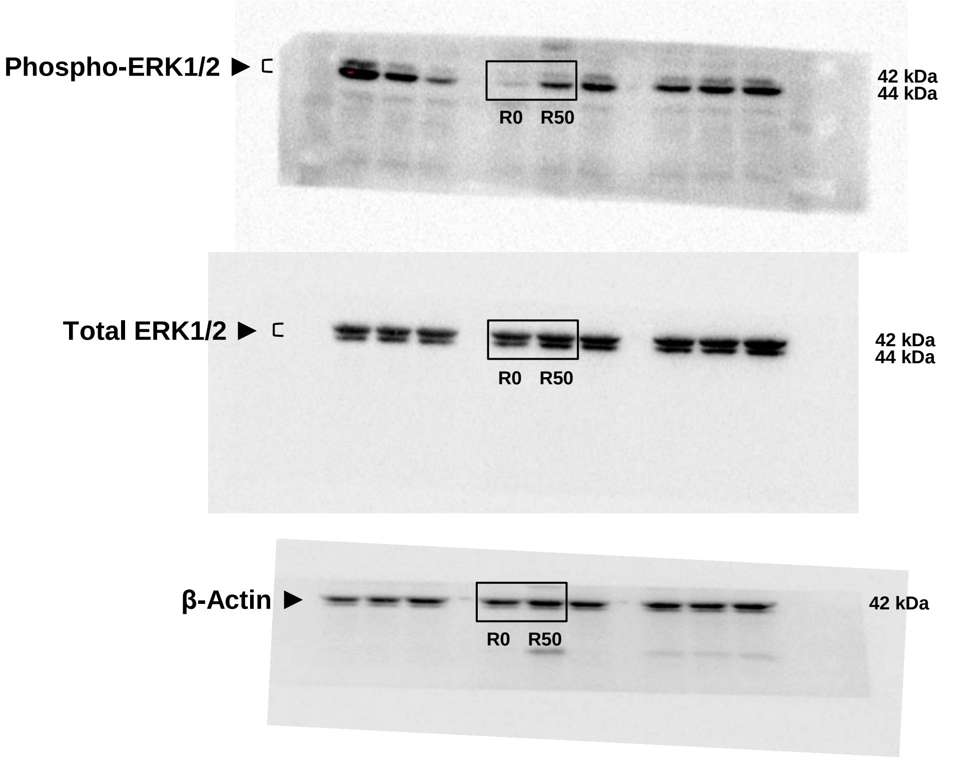

Phospho-ERK1/2
42 kDa
44 kDa
R0
R50
Total ERK1/2
42 kDa
44 kDa
R0
R50
β-Actin
42 kDa
R0
R50
