# Supplementary material for: ADSCs stimulated by resistin promote breast cancer cell malignancy via CXCL5 in a breast cancer coculture model
Source: Sci Rep. 2022 Sep 14;12:15437. doi: 10.1038/s41598-022-19290-6 (PMC9475041; doi:10.1038/s41598-022-19290-6)
Supplement: Supplementary file 3 — Supplementary Information 3. [file 41598_2022_19290_MOESM3_ESM.pdf]

Supplementary Table S1. Clinical characteristics of patient donors for the establishment of adipose-derived stem cell (ADSC) cultures.

Patient donor	#1	#2	#3	#4	#5	#6	#7	#8	#9	#10
Age (y)	65	68	51	78	42	71	70	68	49	62
Stage	IIIA	IIIA	0	nd	IIB	IV	IA	IIIB	IV	IA
Grade	3	2	3	2	2	3	2	3	2	3
Primary tumor	T2	T2	T1	T2	T3	T1	T2	T3	T2	T2
LN metastasis	yes	yes	no	yes	no	yes	no	yes	yes	no
ER status	–	+	–	+	+	+	+	+	+	+
PR status	–	–	–	–	+	+	+	+	+	+
HER2 status	–	+	+	+	+	+	–	+	+	–

The pathologies were classified according to the Cancer Staging Manual of American Joint Committee on Cancer (AJCC; American College of Surgeons, Chicago, IL, USA).
+, positive; –, negative; ER, estrogen receptor; HER2, epidermal growth factor receptor 2; LN, lymph node; nd, not determined; PR, progesterone receptor; T1, tumor size ≤ 2 cm; T2, tumor size > 2 cm but ≤ 5 cm; T3, tumor size > 5 cm.
